# Imeglimin Halts Liver Damage by Improving Mitochondrial Dysfunction in a Nondiabetic Male Mouse Model of Metabolic Dysfunction-Associated Steatohepatitis

**DOI:** 10.3390/antiox13111415

**Published:** 2024-11-18

**Authors:** Kosuke Kaji, Soichi Takeda, Satoshi Iwai, Norihisa Nishimura, Shinya Sato, Tadashi Namisaki, Takemi Akahane, Hitoshi Yoshiji

**Affiliations:** Department of Gastroenterology, Nara Medical University, 840 Shijo-cho, Kashihara 634-8521, Nara, Japan; souitit@naramed-u.ac.jp (S.T.); satoshi181@naramed-u.ac.jp (S.I.); nishimuran@naramed-u.ac.jp (N.N.); shinyasato@naramed-u.ac.jp (S.S.); tadashin@naramed-u.ac.jp (T.N.); stakemi@naramed-u.ac.jp (T.A.); yoshijih@naramed-u.ac.jp (H.Y.)

**Keywords:** imeglimin, hepatocyte, metabolic dysfunction-associated steatohepatitis, fatty acid oxidation, reactive oxygen species, mitochondrial biogenesis

## Abstract

Imeglimin promotes glucose-stimulated insulin secretion in the pancreas in a glucose-dependent manner and inhibits gluconeogenesis in the liver. Meanwhile, imeglimin can improve mitochondrial function in hepatocytes. We used a nondiabetic metabolic dysfunction-associated steatohepatitis (MASH) model to examine the effects of imeglimin on MASH independent of its glucose-lowering action. Mice fed a choline-deficient high-fat diet (CDA-HFD) were orally administered imeglimin (100 and 200 mg/kg twice daily), and MASH pathophysiology was evaluated after 8 weeks. Moreover, an in vitro study investigated the effects of imeglimin on palmitic acid (PA)-stimulated lipid accumulation, apoptosis, and mitochondrial dysfunction in human hepatocytes. CDA-HFD-fed mice showed hepatic steatosis, inflammation, and fibrosis without hyperglycemia. Imeglimin reduced hepatic steatosis in response to increased expression of β-oxidation-related markers. Imeglimin reduced reactive oxygen species accumulation and increased mitochondrial biogenesis in CDA-HFD-fed mice. Consequently, imeglimin suppressed hepatocyte apoptosis and decreased macrophage infiltration with reduced proinflammatory cytokine expression, suppressing hepatic fibrosis development. PA-stimulated hepatocytes induced lipogenesis, inflammatory cytokine production, and apoptosis, which were significantly suppressed by imeglimin. In mitochondrial function, imeglimin improved PA-stimulated decrease in mitochondrial membrane potential, mitochondrial complexes activity, oxygen consumption rate, and mitochondrial biogenesis marker expression. In conclusion, imeglimin could contribute to prevention of MASH progression through suppressing de novo lipogenesis and enhancing fatty acid oxidation.

## 1. Introduction

Metabolic dysfunction-associated steatotic liver disease (MASLD), previously known as nonalcoholic fatty liver disease (NAFLD), is considered a hepatic phenotype of metabolic syndrome, and its prevalence is alarmingly high, affecting >30% of the adult population worldwide [1,2]. The hallmark of MASLD is >5% fat accumulation in the liver upon histological examination [1,2]. This steatosis can progress to subsequent inflammatory metabolic dysfunction-associated steatohepatitis (MASH), followed by advanced fibrosis. Cohorts with patients with MASLD have revealed an overall progression to cirrhosis or end-stage liver disease of about 7–12% over periods of 7–20 years [3,4,5]. Moreover, the yearly cumulative incidence of HCC is approximately 2% in patients with MASH-related cirrhosis, and compared with patients with HCC related to viral hepatitis or alcohol-associated liver disease, those with NAFLD-HCC have a lower male-to-female ratio (1.2:1) [6,7].

Lifestyle-related interventions such as diet and exercise therapies have been recognized to potentially help preventing the progression of MASH [8]. However, effective pharmacological treatments for patients with MASH are still limited, particularly for those without obesity, type 2 diabetes (T2DM), dyslipidemia, and hypertension [4,5]. Vitamin E is particularly recommended for patients with MASH, and evidence from the PIVENS trial showed its beneficial effect on fat deposition, inflammation, and ballooning grade while not on fibrosis resolution [9]. In a recent phase III trial, the thyroid hormone receptor beta-selective agonist resmetirom showed promising results in improving liver fibrosis in patients with MASH. However, it is likely to be some time before this drug becomes available worldwide [10]. Therefore, novel therapies for patients with MASH should be developed urgently.

Among various factors, mitochondrial dysfunction plays a significant role in the pathogenesis of MASH. In MASH, mitochondrial dysfunction impairs fatty acid oxidation and lipid metabolism, resulting in the accumulation of lipids within hepatocytes [7,8,9]. Dysfunctional mitochondria also lead to impaired adenosine triphosphate (ATP) production, contributing to cellular energy deficiency [11,12,13]. This can exacerbate liver damage because hepatocytes require ample energy to perform their functions, including lipid metabolism and detoxification [11,12,13]. Reactive oxygen species (ROS) accumulation by mitochondrial dysfunction can cause hepatocyte death through several mechanisms, such as apoptosis and necrosis [11,12,13,14]. Moreover, dysfunctional mitochondria can trigger inflammatory responses through various pathways, including damage-associated molecular pattern release and inflammatory signaling pathway activation, which contribute to liver fibrosis progression, ultimately leading to cirrhosis [11,12,13,14]. Thus, targeting mitochondrial dysfunction may represent a therapeutic strategy for managing MASH.

Imeglimin, a novel antidiabetic agent, can normalize glucose tolerance and improve insulin sensitivity by targeting mitochondrial bioenergetics [15]. Imeglimin may enhance glucose-stimulated insulin secretion, suppress excessive hepatic gluconeogenesis, inhibit pancreatic beta-cell apoptosis, and maintain pancreatic β-cell mass by preserving or restoring the functional and structural integrity of β-cell mitochondria [16]. In a recent animal study, imeglimin-mediated regulation of glucose homeostasis was shown to be involved in the improvement of hepatic mitochondrial function, leading to favored lipid oxidation and reduced ROS production in high-fat and high-sucrose diet-fed mice [17]. In another cell-based study, imeglimin prevented human endothelial death by decreasing the size of the mitochondrial permeability transition pore, which plays a crucial role in cell death [18]. Moreover, a recent study reported that imeglimin could exhibit pharmacological effects on mitochondrial dysfunction by regulating the gene expression differently to metformin, a classical biguanide drug [19]. However, the effect of imeglimin on liver fibrosis in MASH has not been clarified.

In this study, we examined whether the effects of imeglimin on mitochondrial function could improve MASH pathophysiology. We assessed the effects of imeglimin in a nondiabetic MASH model, focusing on actions independent of the effect of imeglimin on blood glucose.

## 2. Materials and Methods

### 2.1. Animals and Reagents

Six-week-old male C57BL/6J mice (CLEA Japan, Osaka, Japan) were caged with free access to food and water, under controlled temperature (23 °C ± 3 °C) and humidity (50% ± 20%), and a 12 h light/dark cycle. The ethics committee of Nara Medical University (No. 13404) reviewed and approved this study, which was performed following the Guide for Care and Use of Laboratory Animals of the National Research Council. Sumitomo Pharma Co., Ltd. (Osaka, Japan) provided imeglimin hydrochloride.

### 2.2. Animal Treatment

We fed the mice with a choline-deficient, L-amino acid-defined, high-fat diet (CDA-HFD) to induce MASH liver fibrosis for 8 weeks [20], with mice fed a choline-supplemented L-amino acid-defined, normal-fat diet (CSA-NFD) used as the negative control. Both diets were purchased from Research Diets Inc. (New Brunswick, NJ, USA). The mice were randomly divided into five groups (*n* = 10) and treated as follows: (i and ii) fed with CSA-NFD and administered vehicle (i: CS+Veh) or imeglimin (200 mg/kg) (ii: CS+Igm), (iii–v) fed with CDA-HFD and administered vehicle (iii: CD+Veh), imeglimin (100 mg/kg) (iv: CD+Igm-L), or imeglimin (200 mg/kg) (v: CD+Igm-H). Saline was used as the vehicle, and the vehicle and imeglimin were administered with the feeding of CSA-NFD or CDA-HFD by gavage twice daily throughout the study as previously described [17,21,22]. After the experimental period, blood was collected to measure the levels of glucose, aspartate aminotransferase (AST), and alanine aminotransferase (ALT), then the animals were killed using mild isoflurane anesthesia. Whole liver samples were retrieved for biochemical, histopathological, and molecular analyses.

### 2.3. Cell Cultures

HepG2, a human hepatocellular cell line, and LX-2, a human activated hepatic stellate cell (HSC) line, were obtained from the Japanese Collection of Research Bioresources Cell Bank (Osaka, Japan) and Merck KGaA (Darmstadt, Germany), respectively. Short tandem repeat profiling was used to authenticate all cell lines within the last 3 years. Mycoplasma testing was performed using the MycoProbe^®^ Mycoplasma Detection Kit (R&D Systems, Minneapolis, MN, USA) following the manufacturer’s protocol. The cells were cultured in DMEM supplemented with 10% FBS and 1% penicillin–streptomycin at 37 °C under a humidified atmosphere containing 5% CO_2_. To induce steatosis, HepG2 cells (5 × 10^5^ cells) were serum-starved for 18 h and then exposed to palmitic acid (PA) (200 μM; Nacalai Tesque, Kyoto, Japan) for 12 h. Additionally, these cells were treated with different imeglimin concentrations (0, 1, 3, 10, 50 and 100 mM) starting 9 h after the start of PA exposure. Cell viability was determined using The Premix Water-Soluble Tetrazolium salt (WST)-1 Cell Proliferation Assay system (Takara Bio, Kusatsu, Japan) according to the manufacturer’s protocol.

### 2.4. Co-Culture Assay

For co-culture assays, LX-2 cells (1 × 10^5^ cells) were seeded in the lower compartment, whereas HepG2 cells (5 × 10^5^ cells) were cultured in the upper inserts of Transwell™ multiple well plates (24 mm inserts, TC-treated, 0.4 µm pore size, 6-well cluster plate) (Corning, Corning, NY, USA). DMEM containing 2% FBS was used as the co-culture medium. HepG2 cells were incubated with PA for 12 h and further treated with different imeglimin concentrations for the last 3 h.

### 2.5. Histological and Cytological Analyses

Mouse liver samples were fixed in 4% paraformaldehyde and processed into paraffin sections for hematoxylin and eosin and Sirius red staining. Two pathologists independently determined the pathological score to assess liver tissue steatosis, inflammation, and ballooning as previously described [23]. Other liver specimens were fixed with 4% paraformaldehyde for 24 h, then frozen in a cryomold with Tissue-Tek O.C.T compound (Sakura Finetek Japan, Tokyo, Japan) to prepare frozen sections for Oil Red O staining. After the intervention, HepG2 cells were fixed with 4% paraformaldehyde for 25–30 min at room temperature. The cells and frozen liver tissues were stained with Oil Red O dye for 10 min at room temperature.

A TUNEL assay was used to determine apoptotic hepatocytes using an in situ apoptosis detection kit (Takara Bio, Kusatsu, Japan) following the manufacturer’s guidelines. For each sample, the number of positive hepatocytes was counted in five random high-power fields (400× magnification).

For immunohistochemistry, the liver sections were subjected to deparaffinization, rehydration, and immunostaining at 4 °C overnight using primary antibodies (Supplementary Table S1). After washing, the sections were incubated with an anti-rabbit secondary antibody conjugated to horseradish peroxidase (#ab6721; 1:1000, Abcam, Cambridge, UK). The nuclei were counterstained with hematoxylin. For immunofluorescence, mouse monoclonal 4-hydroxynonenal (4-HNE) (as shown in Supplementary Table S1) was used as the primary antibody, and goat anti-mouse IgG H&L (Alexa Fluor^®^ 488) (#ab150113; 1:200; Abcam) was used for detection. Subsequently, the sections were counterstained with 4′,6-diamidino-2-phenylindole. For each sample, the extent of staining was quantified in five random fields (400× magnification) by using ImageJ software version 1.54 (National Institutes of Health, Bethesda, MD, USA).

### 2.6. Biochemical Assays

Hepatic content of triglyceride (TG) and malondialdehyde (MDA) was measured using Triglyceride-Glo™ Assay (Promega, Madison, WI, USA) and OxiSelect^TM^ TBARS Assay Kit (MDA Quantitation) (Cell Biolabs, Inc., San Diego, CA, USA), respectively. The hepatic level of interleukin (IL)-33 and its receptor ST2 was determined using Mouse IL-33 and ST2/IL-33R DuoSet enzyme-linked immunosorbent assay (ELISA) (R&D Systems, Minneapolis, MN, USA). Hepatic hydroxyproline content was measured in frozen mouse liver tissue using the hydroxyproline assay kit (Cell Biolabs, Inc.). The levels of tumor necrosis factor-α (TNF-α), IL-6, and IL-1β in HepG2 cell culture supernatant were measured using human ELISA kits (Abcam). All analyses followed the manufacturer’s recommended protocols.

### 2.7. Cleaved Caspase-3 and Cleaved PARP1 Levels

To evaluate cell apoptosis in mouse liver, cleaved caspase-3 level was measured using human/mouse cleaved caspase-3 (Asp175) DuoSet IC ELISA (R&D Systems) following the manufacturer’s instruction. In vitro cell apoptosis in HepG2 cells was determined as cleaved caspase-3 and cleaved poly (ADP-ribose) polymerase (PARP) 1 concentration in cell extracts using ELISA and FastScan™ cleaved PARP (Asp214) ELISA kit (Cell Signaling Technology, Danvers, MA, USA), respectively, following the manufacturer’s instructions.

### 2.8. RNA Extraction and Real-Time qPCR

Total RNA was isolated from cells and mouse liver tissue by using QIAzole and Qiagen RNeasy Mini Kits (Qiagen, Hilden, Germany), followed by reverse transcription into cDNA using the PrimeScript RT Reagent Kit (TaKaRa, Tokyo, Japan). Subsequently, qPCR was performed using the Power SYBR Green PCR Master Mix (Thermo Fisher Scientific, Waltham, MA, USA) and specified primers detailed in Supplementary Table S2. The 2^−ΔΔCt^ technique was utilized to normalize the target genes’ relative mRNA expression levels to those of the control group and GAPDH concentration.

### 2.9. Protein Extraction and Western Blotting

Total protein extraction commenced with sample treatment using a mammalian protein extraction reagent (Thermo Fisher Scientific) and mixed with Halt™ protease and phosphatase inhibitor cocktail (Thermo Fisher Scientific). Equal quantities of liver tissues and cell-extracted proteins were separated using 10% sodium dodecyl-sulfate polyacrylamide gel electrophoresis and then transferred to an Invitrolon™ polyvinylidene difluoride membrane (Thermo Fisher Scientific). The protein-loaded membranes were blocked with defatted milk (5%) at room temperature for 2 h and incubated overnight with primary antibodies (Supplementary Table S1) at 4 °C. The next day, the membranes were washed and incubated with a horseradish peroxidase-labeled secondary antibody for 1 h at room temperature. Finally, the target bands were detected using chemiluminescence. The loading control was β-actin. All assays were completed at least thrice.

### 2.10. Assessment of Mitochondrial Membrane Potential

The mitochondrial membrane potential was evaluated using tetramethylrhodamine methyl ester (TMRM; FUJIFILM, Wako Pure Chemical Corporation, Osaka, Japan). The HepG2 cells were stained by adding dye to the culture medium at 100 nM for 30 min at 37 °C. Subsequently, the cells were stained using the Hoechst 33342 nuclear dye (Thermo Fisher Scientific) at a 1:500 dilution in PBS at room temperature for 10 min, washed with PBS, and visualized using fluorescence microscopy (BZ-X800; Keyence Corporation, Osaka, Japan). The fluorescence signal intensity was quantified using ImageJ software version 1.54 (National Institutes of Health).

### 2.11. Mitochondrial Respiratory Chain Complex Activity

The mitochondria were isolated from cultured HepG2 cells by homogenization using a Dounce homogenizer (20–30 strokes), followed by low-speed (600× *g*) and high-speed (11,000× *g*) centrifugations using a mitochondria isolation kit for cultured cells (with Dounce homogenizer) (Abcam) following the manufacturer’s instructions. The activity of mitochondrial respiratory chain complex I–V was determined using the MitoCheck Complex I–V Activity Assay Kit (Cayman Chemical, Ann Arbor, MI, USA) following the manufacturer’s protocols.

### 2.12. Mitochondrial Respiration

Oxygen consumption rate (OCR) was measured using the XFe96 extracellular flux analyzer (Agilent Technologies, Inc., Santa Clara, CA, USA) as previously described [24]. Briefly, the HepG2 cells were seeded at 1.5 × 10^5^ per well in a 24-well tissue culture plate for 24 h before running the flux analyzer. To assess the mitochondrial respiration rate, the cells stimulated with/without PA were incubated in an XF base medium (Agilent Technologies, Inc.) supplemented with 10 mM glucose, 1 mM pyruvate, and 2 mM L-glutamine and stimulated with imeglimin for 3 h at 37 °C. Oxygen consumption was measured under basal conditions and after treatment with oligomycin (2 μM), carbonyl cyanide 4-(trifluoromethoxy) phenylhydrazone (FCCP, 1 μM), antimycin A (0.5 μM), and rotenone (0.5 μM) and normalized by protein concentration using TaKaRa BCA protein assay kit (Takara Bio). Basal OCR was calculated as follows: [OCR(initial) − OCR(A + R)]. The maximum OCR was computed as follows: [OCR(FCCP) − OCR(A + R)].

### 2.13. Statistical Analyses

All experimental data were presented as mean ± SD of at least three independent experiments. Statistical significance was analyzed using a two-sided Student’s *t*-test or one-way analysis of variance, followed by Bonferroni’s multiple comparison test, as appropriate, using GraphPad Prism version 9.0 (GraphPad Software, La Jolla, CA, USA). Statistical significance was defined as *p* < 0.05.

## 3. Results

### 3.1. Imeglimin Inhibits Steatohepatitis Progression with Reduced Lipid Accumulation in CDA-HFD-Fed Mice

**Figure 1:**
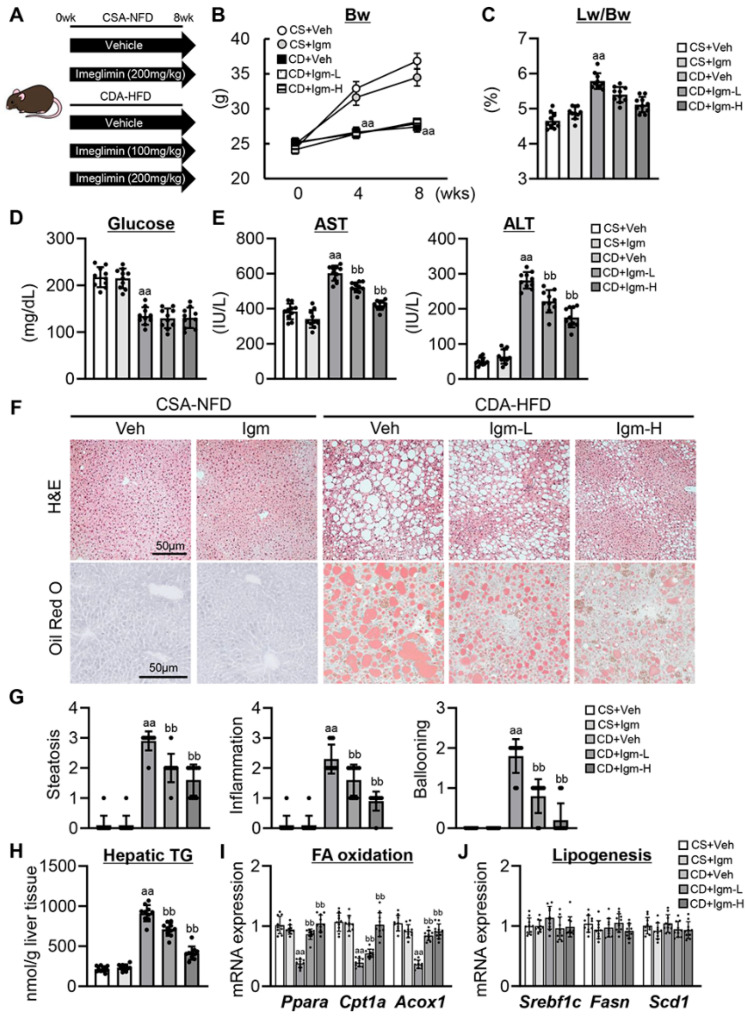
Effect of imeglimin on hepatic steatosis in CDA-HFD-fed mice. (**A**) Mice experimental design. (**B**) Changes in the body weight (Bw) during the experimental period. aa: *p* < 0.01 vs. CS+Veh group, significant difference between groups by Student’s *t*-test. (**C**) Liver/body weight (Lw/Bw) at the end of experiment. aa: *p* < 0.01 vs. CS+Veh group, significant difference between groups by Student’s *t*-test. (**D**) Serum glucose level. aa: *p* < 0.01 vs. CS+Veh group, significant difference between groups by Student’s *t*-test. (**E**) Serum aspartate aminotransferase (AST), alanine aminotransferase (ALT) levels. aa: *p* < 0.01 vs. CS+Veh group, bb: *p* < 0.01 vs. CD+Veh group, significant difference between groups by Student’s *t*-test. (**F**) Representative microphotographs of liver section stained with hematoxylin and eosin (H&E) and Oil Red O. (**G**) Hepatic pathological scores for steatosis, inflammation and ballooning at a 400-fold magnification. aa: *p* < 0.01 vs. CS+Veh group, bb: *p* < 0.01 vs. CD+Veh group, significant difference between groups by Student’s *t*-test. (**H**) Hepatic triglyceride (TG) content. aa: *p* < 0.01 vs. CS+Veh group, bb: *p* < 0.01 vs. CD+Veh group, significant difference between groups by Student’s *t*-test. (**I**,**J**) Hepatic mRNA level of the markers related to (**I**) fatty acid (FA) oxidation and (**J**) lipogenesis. aa: *p* < 0.01 vs. CS+Veh group, bb: *p* < 0.01 vs. CD+Veh group, significant difference between groups by Student’s *t*-test. Gapdh was used as an internal control for qRT-PCR. Quantitative values are indicated as fold changes to the values of CS+Veh group. Data are the mean ± SD (*n* = 10; (**B**–**E**,**G**–**J**)). CS, CSA-NFD-fed mice; CD, CDA-HFD-fed mice; Veh, vehicle-treated group; Igm-L, imeglimin (100 mg/kg)-treated group; Igm-H, imeglimin (200 mg/kg)-treated group.

Figure 1A shows the in vivo experimental design. Eight weeks on the CDA-HFD diet significantly delayed body weight gain and increased relative liver weight. However, these physical impairments were unchanged after treatment with imeglimin (Figure 1B,C). As previously described, the CDA-HFD diet decreased serum glucose levels, indicating that the nondiabetic MASH model and treatment with imeglimin did not alter serum glucose levels (Figure 1D). The serum AST and ALT levels in CDA-HFD-fed mice were higher than those in CSA-NFD-fed mice, and the increases in these indicators were suppressed by treatment with imeglimin at both 100 and 200 mg/kg doses (Figure 1E). The CDA-HFD-fed mice showed typical histological features of MASH, such as hepatic steatosis, inflammation, and ballooning, and these features were attenuated after imeglimin treatment (Figure 1F,G). In CDA-HFD-fed mice treated with imeglimin, attenuation of lipid accumulation was also represented by reduced Oil Red O-stained lipid droplets and hepatic TG levels (Figure 1F,H). In parallel with reduced lipid accumulation, imeglimin treatment ameliorated the CDA-HFD diet-induced decline of hepatic mRNA expression of markers related to fatty acid oxidation, including *Ppara*, *Cpt1a*, and *Acox1* (Figure 1I). Conversely, a trend toward a decrease in hepatic expression related to lipogenesis was noted after treatment with imeglimin in CDA-HFD-fed mice, but the difference was not significant (Figure 1J).

### 3.2. Imeglimin Suppresses ROS Accumulation and Enhances Mitochondrial Biogenesis in the Liver of CDA-HFD-Fed Mice

**Figure 2:**
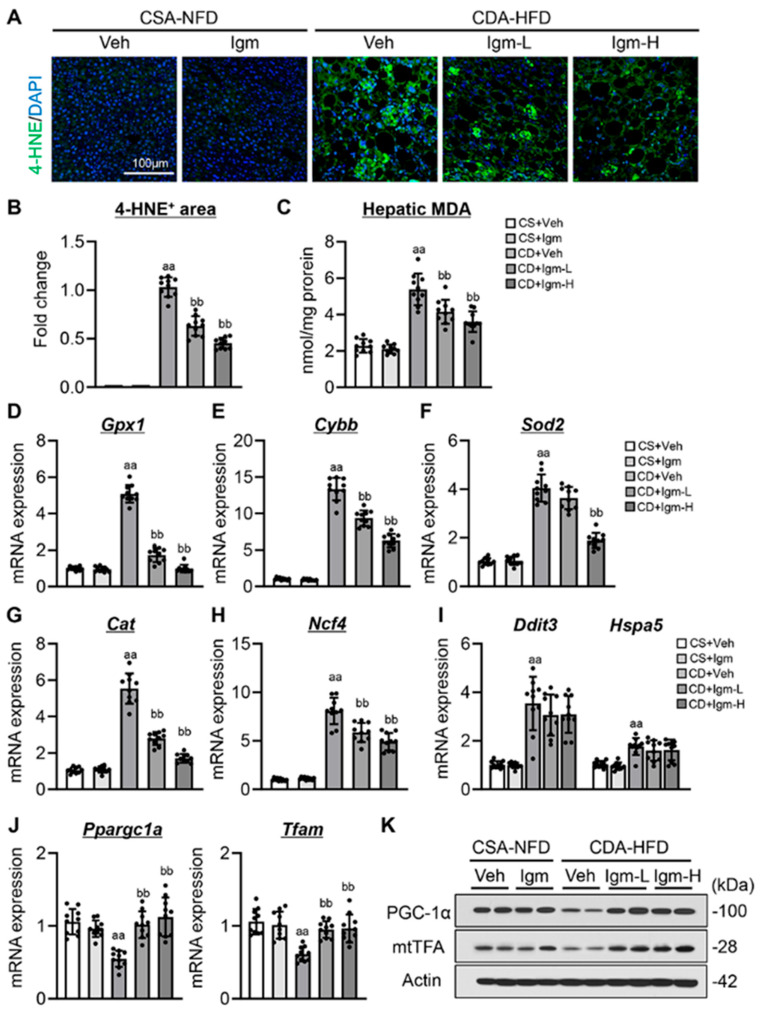
Effect of imeglimin on intracellular stress and mitochondrial biogenesis in CDA-HFD-fed mice. (**A**) Representative microphotographs of liver section immunofluorescent stained with 4-hydroxynonenal (HNE). (**B**) Quantitation of 4-HNE+ area in high-power field (HPF). aa: *p* < 0.01 vs. CS+Veh group, bb: *p* < 0.01 vs. CD+Veh group, significant difference between groups by Student’s *t*-test. (**C**) Hepatic malondialdehyde (MDA) content. aa: *p* < 0.01 vs. CS+Veh group, bb: *p* < 0.01 vs. CD+Veh group, significant difference between groups by Student’s *t*-test. (**D**–**H**) Hepatic mRNA level of five antioxidant enzyme genes, (**D**) Gpx1, (**E**) Cybb, (**F**) Sod2, (**G**) Cat and (**H**) Ncf4. aa: *p* < 0.01 vs. CS+Veh group, bb: *p* < 0.01 vs. CD+Veh group, significant difference between groups by Student’s *t*-test. (**I**) Hepatic mRNA level of ER stress-related markers. aa: *p* < 0.01 vs. CS+Veh group, significant difference between groups by Student’s *t*-test. (**J**) Hepatic mRNA level of mitochondrial biogenesis markers. aa: *p* < 0.01 vs. CS+Veh group, bb: *p* < 0.01 vs. CD+Veh group, significant difference between groups by Student’s *t*-test. (**K**) Western blotting for mitochondrial biogenesis markers. Gapdh and Actin were used as an internal control for qRT-PCR and western blotting, respectively. Quantitative values are indicated as fold changes to the values of CD+Veh (**B**) or CS+Veh group (**D**–**J**). Data are the mean ± SD (*n* = 10; (**B**–**J**)). CS, CSA-NFD-fed mice; CD, CDA-HFD-fed mice; Veh, vehicle-treated group; Igm-L, imeglimin (100 mg/kg)-treated group; Igm-H, imeglimin (200 mg/kg)-treated group.

Considering the suppression of hepatic lipid accumulation, we subsequently evaluated the effect of imeglimin on hepatic ROS production. The CDA-HFD-fed mice showed a marked hepatic expression of 4-hydroxynonenal (4-HNE), an α, β unsaturated hydroxyalkenal, as an oxidative stress biomarker (Figure 2A). Consistently with lipid accumulation, hepatic 4-HNE expression was significantly reduced after imeglimin treatment in the CDA-HFD-fed mice (Figure 2A). Semiquantitative analysis revealed that imeglimin at 200 mg/kg decreased 4-HNE expression by half of vehicle treatment in the CDA-HFD-fed mice (Figure 2B). We confirmed the imeglimin-mediated reduction of hepatic ROS due to decreased levels of hepatic MDA content (Figure 2C). In response to accumulated oxidative stress, the expression levels of antioxidant enzymes (*Gpx1*, *Cybb*, *Sod2*, *Cat*, and *Ncf4*) were increased in CDA-HFD-fed mice (Figure 2D–H). Similarly with hepatic ROS reduction, antioxidant enzyme levels were significantly decreased in the imeglimin-treated groups (Figure 2D–H). Meanwhile, treatment with imeglimin did not affect the increased expression levels of endoplasmic reticulum (ER) stress-related genes (*Ddit3* and *Hspa5*) (Figure 2I). Moreover, the CDA-HFD-fed mice showed decreased mRNA expression of mitochondrial biogenesis markers (*Ppargc1a* and *Tfam*), which were restored after imeglimin treatment (Figure 2J). Moreover, the effect of imeglimin on mitochondrial biogenesis was also observed at protein levels, indicated by increased peroxisome proliferator-activated receptor γ coactivator 1-α (PGC-1α) and mitochondrial transcription factor A (mtTFA) (Figure 2K).

### 3.3. Imeglimin Attenuates Hepatocyte Apoptosis and Macrophage-Mediated Proinflammatory Response in CDA-HFD-Fed Mice

**Figure 3:**
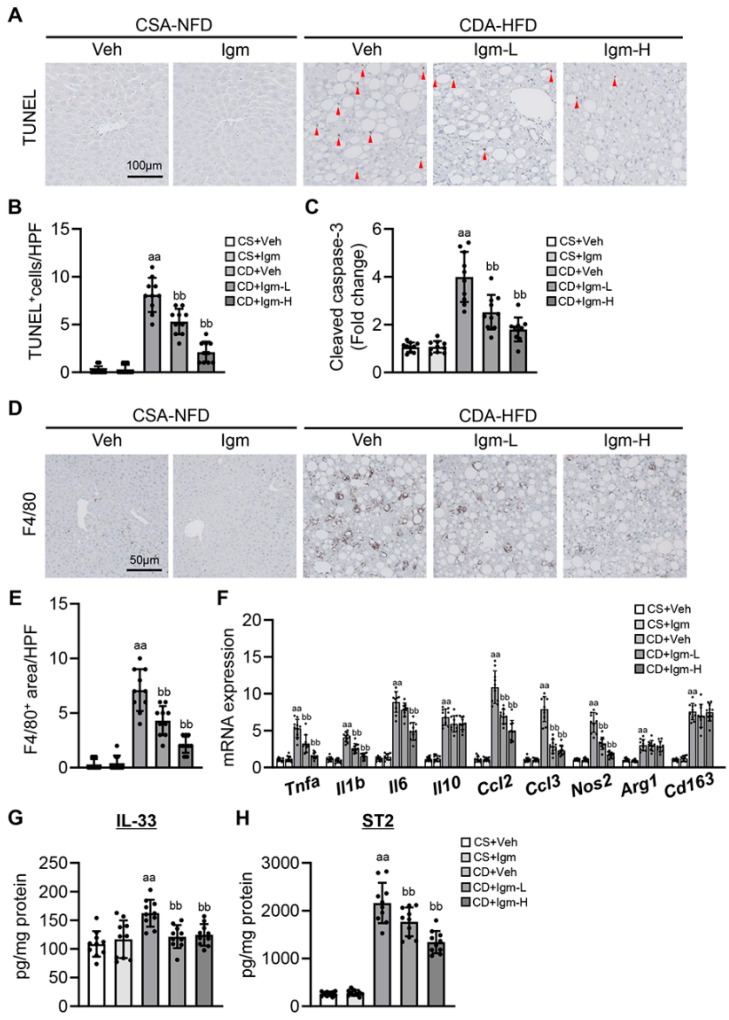
Effect of imeglimin on hepatocyte apoptosis and macrophage infiltration in CDA-HFD-fed mice. (**A**) Representative microphotographs of liver section stained with TUNEL. TUNEL+ hepatocyte indicated with red arrowhead. (**B**) Quantification of TUNEL + hepatocytes in high-power field (HPF). aa: *p* < 0.01 vs. CS+Veh group, bb: *p* < 0.01 vs. CD+Veh group, significant difference between groups by Student’s *t*-test. (**C**) Hepatic level of cleaved caspase-3. aa: *p* < 0.01 vs. CS+Veh group, bb: *p* < 0.01 vs. CD+Veh group, significant difference between groups by Student’s *t*-test. (**D**) Representative microphotographs of liver section stained with F4/80. (**E**) Quantification of F4/80+ macrophages in high-power field (HPF). aa: *p* < 0.01 vs. CS+Veh group, bb: *p* < 0.01 vs. CD+Veh group, significant difference between groups by Student’s *t*-test. (**F**) Hepatic mRNA level of macrophage markers. aa: *p* < 0.01 vs. CS+Veh group, bb: *p* < 0.01 vs. CD+Veh group, significant difference between groups by Student’s *t*-test. (**G**,**H**) Hepatic level of (**G**) IL-33 and (**H**) its receptor ST2. aa: *p* < 0.01 vs. CS+Veh group, bb: *p* < 0.01 vs. CD+Veh group, significant difference between groups by Student’s *t*-test. Gapdh was used as an internal control for qRT-PCR. Quantitative values are indicated as fold changes to the values of CS+Veh group (**C**,**F**). Data are the mean ± SD (*n* = 10; (**B**,**C**,**E**–**H**)). CS, CSA-NFD-fed mice; CD, CDA-HFD-fed mice; Veh, vehicle-treated group; Igm-L, imeglimin (100 mg/kg)-treated group; Igm-H, imeglimin (200 mg/kg)-treated group.

In CDA-HFD-fed mice, TUNEL-positive apoptotic hepatocytes were markedly increased with ROS accumulation (Figure 3A,B). Hepatocyte apoptosis in CDA-HFD mice was also indicated by elevated cleaved caspase-3 levels in the liver tissue (Figure 3C), but they were significantly suppressed after imeglimin treatment (Figure 3A–C). Subsequently, we evaluated the effect of imeglimin on the inflammatory status in the liver of CDA-HFD-fed mice. Treatment with imeglimin reduced the hepatic infiltration of F4/80-positive macrophages, which were extensively observed in CDA-HFD-fed mice (Figure 3D,E). CDA-HFD-fed mice showed increased hepatic mRNA levels of both markers related to M1 (*Tnfa*, *Il1b*, *Il6*, *Ccl2*, *Ccl3*, and *Nos2*) and M2 macrophages (*Il10*, *Arg1*, and *Cd163*) (Figure 3F). Notably, imeglimin suppressed marker expression mainly in M1 macrophages, whereas it had almost no effect on M2 macrophage markers (Figure 3F). In a recent study, mitochondrial DNA released from injured hepatocytes under lipotoxicity induced the upregulation of IL-33 expression in macrophages and enhanced lipopolysaccharide (LPS)-induced inflammatory cytokine production [25]. Imeglimin significantly reduced the hepatic levels of IL-33 and its receptor, ST2, in CDA-HFD-fed mice (Figure 3G,H).

### 3.4. Imeglimin Ameliorates Hepatic Fibrosis Development in CDA-HFD-Fed Mice

**Figure 4:**
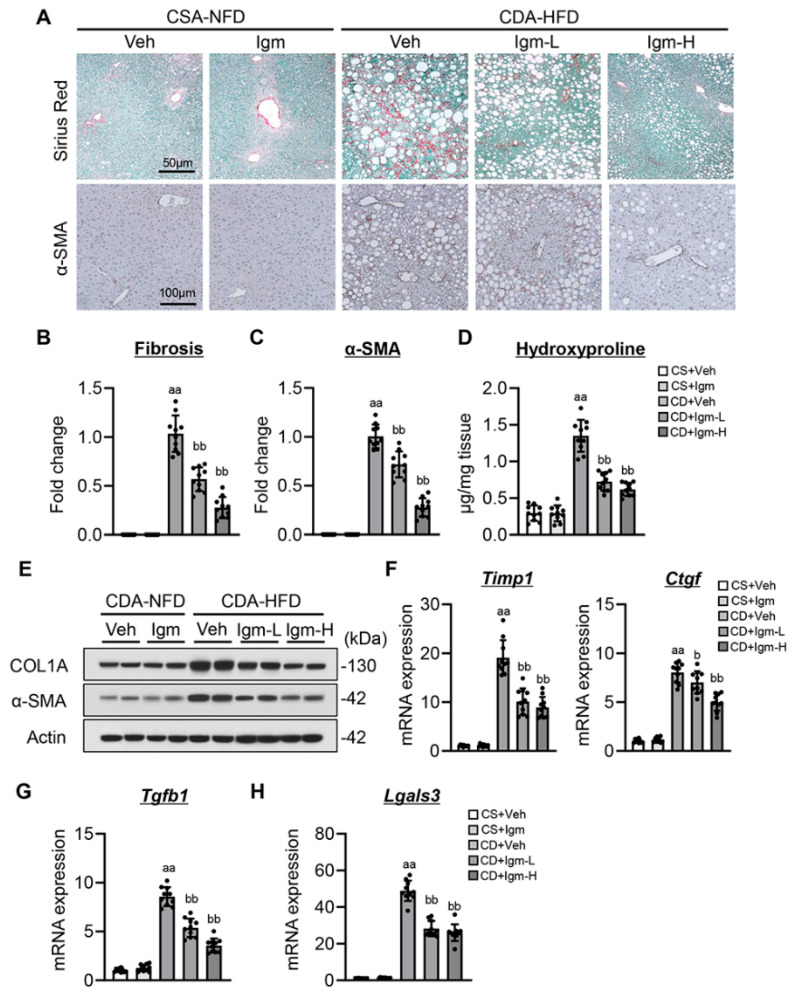
Effect of imeglimin on hepatic fibrosis in CDA-HFD-fed mice. (**A**) Representative microphotographs of liver section stained with Sirius red and α-smooth muscle actin (SMA). (**B**,**C**) Quantification of (**B**) Sirius red stained fibrotic area and (**C**) α-SMA-positive area in high-power field (HPF). aa: *p* < 0.01 vs. CS+Veh group, bb: *p* < 0.01 vs. CD+Veh group, significant difference between groups by Student’s *t*-test. (**D**) Hepatic content of hydroxyproline. aa: *p* < 0.01 vs. CS+Veh group, bb: *p* < 0.01 vs. CD+Veh group, significant difference between groups by Student’s *t*-test. (**E**) Western blotting for protein expression of COL1A and α-SMA. (**F**–**H**) Hepatic mRNA level of (**F**) Timp1 and Ctgf, (**G**) Tgfb1, and (**H**) Lgals3. aa: *p* < 0.01 vs. CS+Veh group, b,bb: *p* < 0.05, 0.01 vs. CD+Veh group, significant difference between groups by Student’s *t*-test. Gapdh and Actin were used as an internal control for qRT-PCR and western blotting, respectively. Quantitative values are indicated as fold changes to the values of CD+Veh (**B**,**C**) or CS+Veh (**F**–**H**). Data are the mean ± SD (*n* = 10; (**B**–**D**,**F**–**H**)). CS, CSA-NFD-fed mice; CD, CDA-HFD-fed mice; Veh, vehicle-treated group; Igm-L, imeglimin (100 mg/kg)-treated group; Igm-H, imeglimin (200 mg/kg)-treated group.

CDA-HFD-fed mice developed significant pericellular fibrosis with HSC activation as evaluated by Sirius red and α-smooth muscle actin (α-SMA) staining, whereas treatment with imeglimin improved CDA-HFD feeding-induced hepatic fibrosis development (Figure 4A–C). Consistent with fibrosis attenuation, the hepatic content of hydroxyproline and protein expressions of collagen type I alpha 1 (COL1A) and α-SMA were significantly reduced after imeglimin treatment in CDA-HFD-fed mice (Figure 4D,E). The observed imeglimin-mediated attenuation of hepatic fibrosis coincided with decreased hepatic expression levels of genes related to fibrogenesis, including *Timp1*, *Ctgf*, and *Tgfb1* (Figure 4F,G). Moreover, galectin-3 has a profibrotic role by interplaying with the IL-33/ST2 pathway in diet-induced steatohepatitis [26]. Treatment with imeglimin suppressed the hepatic upregulation of *Lgals3* coding for galectin-3 in CDA-HFD-fed mice along with decreased IL-33 and ST2 levels (Figure 4H).

### 3.5. Effect of Imeglimin on PA-Stimulated Lipotoxicity, Inflammation, and Apoptosis in Human Hepatocytes

**Figure 5:**
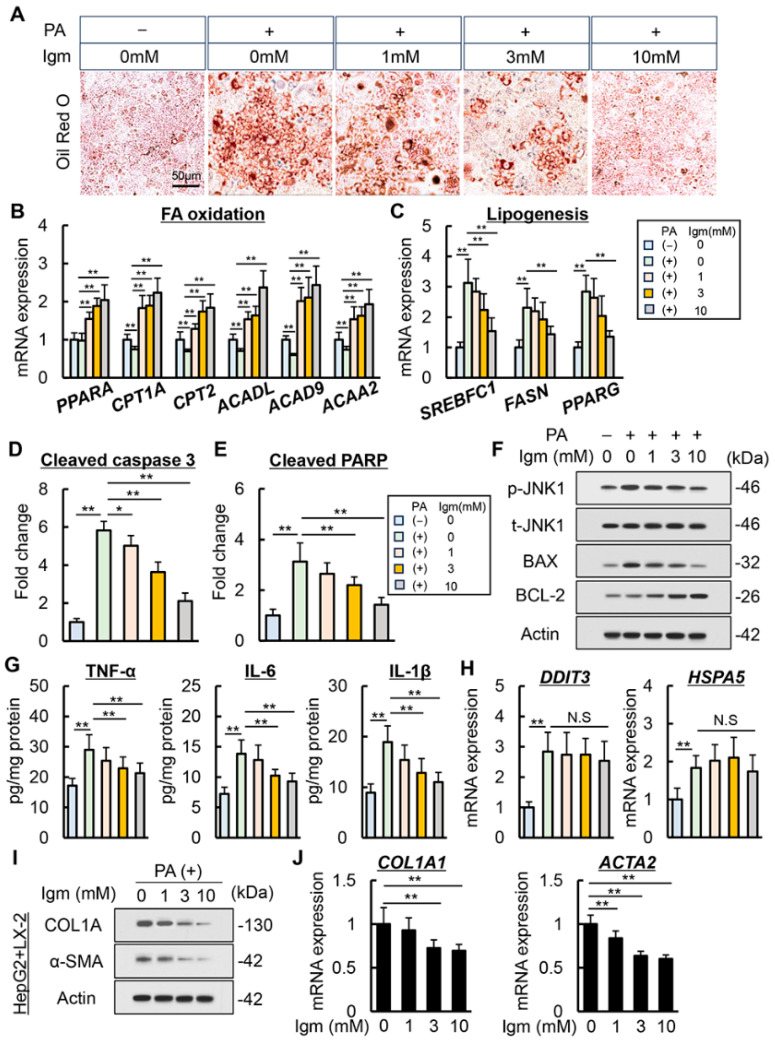
Effect of imeglimin on palmitic acid-stimulated lipid accumulation and apoptosis in human hepatocyte. (**A**) Representative microphotographs of palmitic acid (PA)-stimulated HepG2 stained with Oil Red O. (**B**,**C**) Intracellular mRNA expression of (**B**) fatty acid (FA) oxidation-related genes and (**C**) lipogenesis-related genes in PA-stimulated HepG2. **: *p* < 0.01, significant difference between groups by Student’s *t*-test. (**D**,**E**) Intracellular level of (**D**) cleaved caspase-3 and (**E**) cleaved PARP in PA-stimulated HepG2. *: *p* < 0.05, **: *p* < 0.01, significant difference between groups by Student’s *t*-test. (**F**) Western blotting for phospho-JNK (p-JNK), total JNK (t-JNK), BAX and BCL-2 expression in PA-stimulated HepG2. (**G**) Intracellular level of TNF-α, IL-6 and IL-1β in PA-stimulated HepG2. **: *p* < 0.01, significant difference between groups by Student’s *t*-test. (**H**) Intracellular mRNA expression of ER stress-related genes in PA-stimulated HepG2. **: *p* < 0.01, significant difference between groups by Student’s *t*-test. (**I**,**J**) Intracellular (**I**) protein and (**J**) mRNA expression of profibrogenic markers in LX-2 co-cultured with PA-stimulated HepG2. **: *p* < 0.01, significant difference between groups by Student’s *t*-test. HepG2 cells were treated with 0.5 mM of PA and 0, 1, 3, 10 mM of imeglimin under both mono-culture and co-coculture. Gapdh and Actin were used as an internal control for qRT-PCR and western blotting, respectively. Quantitative values are indicated as fold changes to the values of PA (−)/Igm 0 mM-treated group (**B**–**E**,**H**,**J**). Data are the mean ± SD (*n* = 8; (**B**–**E**,**G**,**H**,**J**)). N.S, not significant.

To further assess the pharmacological action of imeglimin on hepatocytes under the pathogenesis of MASH, we analyzed its effect on PA-stimulated HepG2 cells. Cell viability assays showed that addition of imeglimin at below 10 mM did not affect the viability of HepG2 cells, while 50 and 100 mM were cytotoxic (Supplementary Figure S1). Therefore, concentrations of 0, 1, 3, and 10 mM were used in subsequent studies. PA stimulation induced lipid accumulation as evaluated with Oil Red O staining in HepG2 cells, which was reduced by treatment with imeglimin in a dose-dependent manner (Figure 5A). Imeglimin significantly increased the intracellular expression of genes related to fatty acid oxidation (*PPARA*, *CPT1A*, *CPT2*, *ACADL*, *ACAD9*, and *ACAA2*) in PA-exposed HepG2 cells, which was consistent with the results in the MASH mouse model (Figure 5B). In addition, imeglimin attenuated gene upregulation related to lipogenesis (*SREBFC1*, *FASN*, and *PPARG*) (Figure 5C). In accordance with lipotoxicity reduction, imeglimin treatment decreased the intracellular levels of cleaved caspase-3 and cleaved PARP, indicating the inhibition of PA-induced apoptosis in HepG2 cells (Figure 5D,E). Western blot analysis showed decreased p-c-Jun N-terminal kinase 1 (JNK1) and Bcl-2-associated X protein (BAX) expression and increased B-cell/CLL lymphoma 2 (BCL-2) expression after imeglimin treatment (Figure 5F). Imeglimin also reduced the production of inflammatory cytokines, including TNF-α, IL-6, and IL-1β, elevated by PA stimulation in HepG2 cells (Figure 5G). Meanwhile, imeglimin did not affect the expression of ER stress-related markers, consistent with the results of in vivo experiments (Figure 5H). Subsequently, we assessed the effect of imeglimin on activated HSCs using LX-2 cells. Imeglimin did not affect the profibrogenic marker expression levels in monocultured LX-2 cells (Supplementary Figure S2). By contrast, in LX-2 cells co-cultured with HepG2 cells, imeglimin significantly reduced COL1A and α-SMA protein expression and mRNA expression levels (Figure 5I,J).

### 3.6. Effect of Imeglimin on PA-Stimulated Mitochondrial Dysfunction in Human Hepatocytes

**Figure 6:**
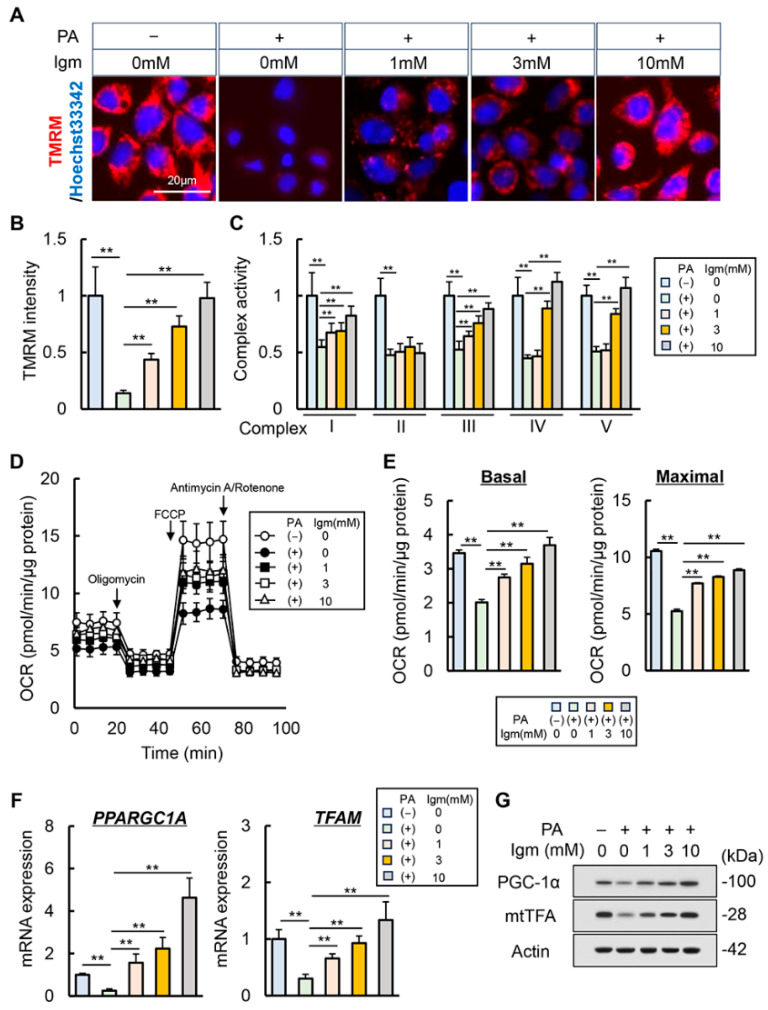
Effect of imeglimin on mitochondrial dysfunction in palmitic acid-stimulated human hepatocyte. (**A**) Representative images of TMRM live stains corresponding to mitochondrial membrane potential in palmitic acid (PA)-stimulated HepG2. (**B**) Quantification of TMRM intensity per cell; data shown as the mean ± SD for 50 cells per condition in three representative experiments. Nuclei were stained with Hoechst 33342. **: *p* < 0.01, significant difference between groups by Student’s *t*-test. (**C**) Relative activity of mitochondrial respiratory chain complex I–V in PA-stimulated HepG2. **: *p* < 0.01, significant difference between groups by Student’s *t*-test. (**D**) Measurements of oxygen consumption rate (OCR) using a seahorse extracellular flux analyzer. (**E**) Calculations of the basal and maximal respiration rates. **: *p* < 0.01, significant difference between groups by Student’s *t*-test. (**F**,**G**) Intracellular (**F**) mRNA and (**G**) protein expression of mitochondrial biogenesis markers in PA-stimulated HepG2. **: *p* < 0.01, significant difference between groups by Student’s *t*-test. HepG2 cells were treated with 0.5 mM of PA and 0, 1, 3, 10 mM of imeglimin. Gapdh and Actin were used as an internal control for qRT-PCR and western blotting, respectively. Quantitative values are indicated as fold changes to the values of PA (−)/Igm 0 mM-treated group (**B**,**C**,**F**). Data are the mean ± SD (*n* = 8; (**B**–**F**)).

The effect of imeglimin on PA-stimulated mitochondrial dysfunction in HepG2 cells was examined. Mitochondrial membrane potential, a mitochondrial function indicator, was assessed using the fluorescence intensity of TMRM that accumulates in the mitochondria. The mitochondrial membrane potential was decreased in PA-stimulated HepG2 cells, which were restored after imeglimin treatment (Figure 6A,B). Subsequently, we measured the mitochondrial complex (complex I–V) activities, which are responsible for oxidative phosphorylation-mediated mitochondrial ATP production. In PA-stimulated HepG2 cells, all complex I–V activities were significantly decreased compared with HepG2 cultured in normal conditions (Figure 6C). Notably, imeglimin treatment effectively recovered the activity of complex I, III, IV, and V in PA-stimulated HepG2 cells, although it had no significant effect on complex II activity (Figure 6C). Subsequently, the cellular effects of imeglimin on mitochondrial respiration were investigated using seahorse analyses to measure the OCR of HepG2 cells stimulated with PA. The OCR values were normalized to the cellular mitochondrial content, and the basal and maximal respiration rates were calculated. Our findings showed that PA stimulation downregulated basal OCR and maximal respiratory capacity in HepG2 cells, which were restored after imeglimin treatment (Figure 6D,E). Moreover, we evaluated the effect of imeglimin on mitochondrial biogenesis. Stimulation with PA in HepG2 cells decreased the intracellular mRNA levels of *PPARGC1A* and *TFAM* (Figure 6F). The expression of markers related to mitochondrial biogenesis was decreased at the protein level, and imeglimin restored these expression levels (Figure 6G).

## 4. Discussion

To the best of our knowledge, our study demonstrated that imeglimin could inhibit hepatic steatosis, oxidative stress, and hepatocyte apoptosis, resulting in the amelioration of liver fibrosis progression in a nondiabetic MASH model. We used the CDA-HFD-fed mouse model to create a model of nondiabetic MASH with advanced fibrosis progression in rodents. Chronic feeding of the CDA-HFD rapidly leads to hepatic fibrosis progression within 6–9 weeks; thus, it is potentially useful for mimicking human MASLD [20]. Notably, this model developed steatohepatitis without hyperglycemia and insulin resistance [27]. Moreover, the CDA-HFD causes mitochondrial dysfunction with oxidative stress in the liver [28]. Considering these properties of the CDA-HFD, the present mouse model seems highly suitable for investigating the effect of imeglimin on MASH under nondiabetic conditions.

Our findings revealed that imeglimin normalized fatty acid oxidation in the liver of CDA-HFD-fed mice and PA-stimulated HepG2 cells. Impaired fatty acid oxidation can cause intracellular free fatty acid accumulation, exacerbate lipotoxicity, and ultimately induce inflammation [11,12,13,14,29,30]. In MASH, mitochondrial ROS accumulation plays a negative role in mitochondrial oxidative reactions and impaired fatty acid oxidation, ultimately exacerbating lipid substance accumulation and toxic by-product formation [11,12,13,14,29,30]. In the present murine and cell-based studies, imeglimin did not alter glycemic status, indicating that the effect of imeglimin on hepatocyte fatty acid oxidation is mediated at least in part through mitochondrial action. Regarding lipogenesis, our results showed that imeglimin decreased lipogenesis in PA-stimulated HepG2 cells. PA stimulation increases the expression of sterol regulatory element binding protein 1, a master regulator of lipogenesis, by inhibiting 5′ adenosine monophosphate-activated protein kinase (AMPK) activity [31,32]. In a recent study, imeglimin, similar to metformin, was shown to activate AMPK, which may be involved in the mechanism for inhibition of lipogenesis by imeglimin in PA-stimulated HepG2 cells [19]. Conversely, lipogenesis does not occur in CDA-HFD-fed mice, and assessment of the effect of imeglimin is difficult [20,33]. Therefore, in vivo studies using other models are needed.

Our results showed that, in accordance with increased fatty acid oxidation, imeglimin treatment decreased ROS accumulation and hepatocyte apoptosis, whereas ER stress was not altered. Recent evidence showed the key role that the interaction between ER and oxidative stress plays in the pathogenesis of MASLD [34,35,36]. Particularly, the mitochondria-associated membrane serves as a structural bridge for the functional accumulation of molecules, especially for the exchange of Ca^2+^, lipids, and ROS [37,38]. Thus, further studies are required to discuss the findings that improved mitochondrial function in hepatocytes by imeglimin did not have a significant effect on ER stress.

To approach the glucose-independent effect of imeglimin, we focused on its action on mitochondrial function. Because imeglimin restored the mitochondrial biogenesis in the liver of CDA-HFD-fed mice, we assessed the potent protective effect against PA-stimulated mitochondrial dysfunction in hepatocytes, which was indicated by the retention of mitochondrial membrane potential. The generation of mitochondrial membrane potential is regulated by the mitochondrial respiratory chain, which catalyzes a series of redox reactions that link electron flux with the vectorial transfer of protons from the matrix to the intermembrane space [39,40]. The reaction begins at complex I, where electrons are passed from nicotinamide adenine dinucleotide (NADH) to ubiquinone and then to complexes III and IV, ultimately reducing oxygen to H_2_O. The established mitochondrial membrane potential is used to synthesize ATP from adenosine diphosphate (ADP) and phosphate. Several studies have reported the effects of imeglimin on the mitochondrial respiratory chain in the liver. In a study using high-fat and high-sucrose diet-fed mice, imeglimin was shown to inhibit complex I and restore complex III activities in mouse liver mitochondria [17]. Meanwhile, imeglimin has been shown to have a limited effect on complex I activity in rat hepatocytes, unlike metformin [41]. Hozumi et al. also demonstrated that imeglimin could upregulate the gene coding complexes I (ND1, 2, and 3) and III (CYTB) in HepG2 and primary mice hepatocytes [19]. Notably, our results showed that imeglimin restored complex I, III, and IV activities in PA-exposed HepG2 cells. These findings demonstrate that imeglimin may help protect against electron transport chain impairment with NADH as an electron donor, resulting in the augmentation of a membrane potential to synthesize ATP from ADP. Moreover, our results indicated that imeglimin also recovered the activity of complex V, a mitochondrial ATP synthetase that generates ATP from ADP in the mitochondrial matrix using the energy from the proton electrochemical gradient [39,40,42]. Consequently, imeglimin improved oxygen respiratory function and mitochondrial biogenesis, which was impaired by PA exposure. The present study revealed that imeglimin efficiently ameliorated PA-stimulated mitochondrial dysfunction in hepatocytes, but the mechanism of how imeglimin regulates mitochondrial complex activity in hepatocytes is unclear and considered a subject for future investigation.

Our study has several limitations. First, this study demonstrated the preventive effects of imeglimin on the progression of CDA-HFD-induced steatohepatitis. However, questions surrounding curative effect of imeglimin against established liver fibrosis remain. To this end, further study is required by switching to a standard diet with or without imeglimin, or maintenance on the CDA-HFD diet with or without imeglimin. Moreover, additional detailed studies are needed on the differential effects of imeglimin according to gender and comparison with exercise therapy [43,44]. Second, although our in vitro study used HepG2 cells as human hepatocytes, these cells are derived from hepatoblastoma and not actually replicating human hepatocytes. Various reports have used HepG2 cells to evaluate the pharmacological effect on lipid metabolism and mitochondrial function in hepatocytes during the progression of MASLD and MASH [45,46,47]. Thus, for the purposes of this study, the use of HepG2 cells as hepatocytes is partially suitable on the basis of previous reports. However, primary human hepatocytes should be used to determine whether imeglimin has a similar effect. Third, the CDA-HFD-fed mouse model does not fully recapitulate the pathology of MASH patients, and other models may need to be validated, particularly with regard to the assessment of metabolic disturbances [48]. Moreover, imeglimin was administered to mice twice a day in this study. Thus, there is a concern about the possibility that oral gavage could stress mice and affect their metabolism by fluctuating cortisol. Additionally, we need to consider the impact of the liver circadian clock regulating several physiological functions in the liver, including lipid metabolism, mitochondrial activity, and fibrosis pathway [49,50].

In conclusion, imeglimin may prevent the development of hepatic steatosis, apoptosis, inflammation, and fibrosis in CDA-HFD-induced MASH mice. Notably, imeglimin improved mitochondrial dysfunction, leading to augmented β-oxidation and reduced ROS accumulation in hepatocytes of MASH liver even under nondiabetic conditions. Moreover, the results from our in vivo study showed that a low dose of imeglimin (100 mg/kg) exerted a preventive effect on MASH progression as effectively as 200 mg/kg (the dose used in various rodent experiments examining its antidiabetic effect). These findings suggest that this agent may be clinically viable in ensuring safety in patients with MASH. Given that imeglimin is safe, our results indicated that it may eventually emerge as a viable treatment option for MASH.

## Data Availability

The original contributions presented in the study are included in the article/Supplementary Materials, further inquiries can be directed to the corresponding author.

## Supplementary Materials

The following supporting information can be downloaded at: https://www.mdpi.com/article/10.3390/antiox13111415/s1, Figure S1: Cell viability by treatment with imeglimin in palmitic acid-stimulated HepG2 cells. Figure S2: Imeglimin had no direct effect on activated hepatic stellate cells. Table S1: List of primary antibodies. Table S2: List of primers used in q-PCR.

## Abbreviations

ADP, adenosine diphosphate; ALT, alanine aminotransferase; AMPK, 5′ adenosine monophosphate-activated protein kinase; AST, aspartate aminotransferase; ATP, adenosine triphosphate; BAX, Bcl-2-associated X protein; BCL-2, B-cell/CLL lymphoma 2; CDA-HFD, choline-deficient, L-amino acid-defined, high-fat diet; COL1A, collagen type I alpha 1; CSA-NFD, choline-supplemented L-amino acid-defined, normal-fat diet; ELISA, enzyme-linked immunosorbent assay; ER, endoplasmic reticulum; FCCP, Carbonyl cyanide 4-(trifluoromethoxy) phenylhydrazone; HCC, hepatocellular carcinoma; 4-HNE, 4-hydroxynonenal; HSC, hepatic stellate cell; IL, interleukin; JNK, c-Jun N-terminal kinase; LPS, lipopolysaccharide; MDA, malondialdehyde; MASH, metabolic dysfunction-associated steatohepatitis; MASLD, metabolic dysfunction-associated steatotic liver disease; mtTFA, mitochondrial transcription factor A; NADH, nicotinamide adenine dinucleotide; NAFLD, nonalcoholic fatty liver disease, OCR, oxygen consumption rate; PA, palmitic acid; PARP, poly [ADP-ribose] polymerase 1; PGC-1α, peroxisome proliferator-activated receptor γ coactivator 1-α; ROS, reactive oxygen species; α-SMA, α-smooth muscle actin; TG, triglyceride; TMRM, tetramethylrhodamine methyl ester; TNF-α, tumor necrosis factor-α; T2DM, type 2 diabetes mellitus.
