# Supplementary material for: Imeglimin Halts Liver Damage by Improving Mitochondrial Dysfunction in a Nondiabetic Male Mouse Model of Metabolic Dysfunction-Associated Steatohepatitis
Source: Antioxidants (Basel). 2024 Nov 18;13(11):1415. doi: 10.3390/antiox13111415 (PMC11591211; doi:10.3390/antiox13111415)
Supplement: Supplementary file 1 [file antioxidants-13-01415-s001.zip › antioxidants-3217635-supplementary.pdf]

Supplementary materials

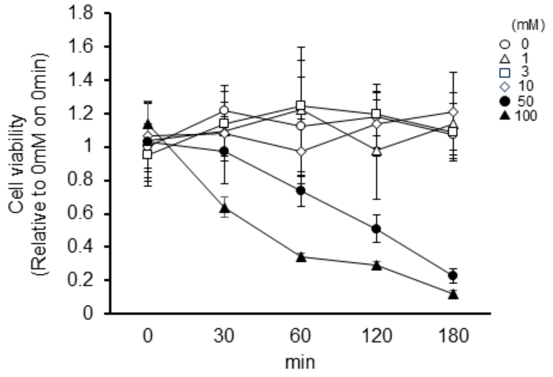

Supplementary Figure S1. Cell viability by treatment with imeglimin in palmitic acid-stimulated HepG2 cells. HepG2 cells were treated with different imeglimin concentrations (0, 1, 3, 10, 50 and 100 mM) starting 9 h after the start of palmitic acid exposure. Cell viability was determined using The Premix Water-Soluble Tetrazolium salt (WST)-1 Cell Proliferation Assay system. Quantitative values are indicated as fold changes to the values of imeglimin 0mM-treated group on 0 min. Data are the mean ± SD (n = 8).

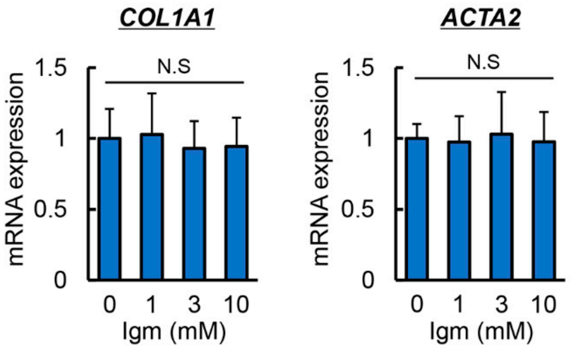

Supplementary Figure S2. Imeglimin had no direct effect on activated hepatic stellate cells. Intracellular mRNA expression of *COL1A1* and *ACTA2* in cultured LX-2 cells. LX-2 cells were treated with 0, 1, 3, 10 mM of imeglimin. *GAPDH* was used as an internal control for qRT-PCR. Quantitative values are indicated as fold changes to the values of Igm 0mM-treated group. Data are the mean ± SD (n = 8). N.S, not significant.

Supplementary Table S1 List of primary antibodies

Antibody	Source (catalog number)	Application (Dilution)
**mouse**		
4-HNE	JaICA (MHN-100P)	IHC (1:4)
PGC-1α	Abcam (ab188102)	WB (1:1000)
mtTFA	Abcam (ab47517)	WB (1:1000)
F4/80	Abcam (ab111101)	IHC (1:100)
α-SMA	Abcam (ab5694)	IHC (1:50), WB (1:100)
COL1A	Santa Cruz (sc-59772)	WB (1:1000)
Actin	Cell Signaling (4967)	WB (1:1000)
**Human**		
p-JNK1$^{(Thr183)}$	Abcam (ab47337)	WB (1:1000)
JNK1	Abcam (ab199380)	WB (1:1000)
BAX	Cell Signaling (2772)	WB (1:1000)
BCL-2	Abcam (ab182858)	WB (1:2000)
α-SMA	Abcam (ab5694)	WB (1:100)
COL1A	Santa Cruz (sc-59772)	WB (1:1000)
PGC-1 α	Abcam (ab188102)	WB (1:1000)
mtTFA	Abcam (ab47517)	WB (1:1000)
Actin	Cell Signaling (4967)	WB (1:1000)

Supplementary Table S2 List of primers used in q-PCR

gene	Sense (5'-3')	Antisense (5'-3')
**Mouse**		
Ppara	ATGCCAGTACTGCCGTTTTC	TTGCCCAGAGATTTGAGGTC
Cpt1a	ATGCTTCTCGCTGTTGGTGA	GTCAGCTGGTAGTGGTGTTG
Acox1	ATGGCTCTCGTGTGCTATCC	TGGGACTCTGTCGTTGGTAG
Srebf1c	CGACTACATCCGCTTCTTGCAG	CCTCCATAGACACATCTGTGCC
Fasn	CTGAGATCCCAGCACTTCTTGA	GCCTCCGAAGCCAAATGAG
Scd1	TTCTTGCGATACACTCTGGTGC	CGGGATTGAATGTTCTTGTCGT
Gpx1	CGCTCTTTACCTTCCTGCGGAA	AGTTCCAGGCAATGTCGTTGCG
Cybb	TGGCGATCTCAGCAAAAGGTGG	GTACTGTCCCACCTCCATCTTG
Sod2	TAACGCGCAGATCATGCAGCTG	AGGCTGAAGAGCGACCTGAGTT
Cat	CGGCACATGAATGGCTATGGATC	AAGCCTTCCTGCCTCTCCAACA
Ncf4	ACTGGAGCTTCTGGTTCTGG	AAGTCTGGGCTGCTTGTGTC
Ddit3	CTGGAAGCCTGGTATGAGGA	TGGTGCTTGGTGAGTGAGA
Hspa5	AGATGAAGGAGGAGGAGGAGGA	AGTGGGTACAGATAGGGGGTG
Ppargc1a	GAATCAAGCCACTACAGACACCG	CATCCCTCTTGAGCCTTTCGTG
Tfam	GAGGCAAAGGATGATTCGGCTC	CGAATCCTATCATCTTTAGCAAGC
Tnfa	CAGGCGGTGCCTATGTCTC	CGATCACCCCGAAGTTCAGTAG
Il6	TACCACTTCACAAGTCGGAGGC	CTGCAAGTGCATCATCGTTGTTC
Il1b	TGGACCTTCCAGGATGAGGACA	GTTCATCTCGGAGCCTGTAGTG
Il10	TGCACTACCAAAGCCACAAG	TCAGTAAGAGCAGGCAGCAT
Ccl2	AGGTCCCTGTCATGCTTCTG	TCTGGACCCATTCCTTCTTG
Ccl3	TGGCTCAGCCAGATGCAATC	AGGCTCCCAGGTCTCTTTGG
Nos2	CCTTGTTCAGCTACGCCTTC	CTTCAGAGTCTGCCCATTGC
Arg1	GCAGTTGGAAGCATCTCTGG	GAGAAAGGACACAGGTTGCC
Cd163	ATGGGTGGACACAGAATGGT	AGCTCACAGCCACAACAAAG
Timp1	CATGGAAAGCCTCTGTGGATATG	GATGTGCAAATTTCCGTTCCTT
Ctgf	TGCCATGAGGAGTGGGTGTG	TGGAGATTTTGGGAGTACGGG
Tgfb1	TTGCTTCAGCTCCACAGAGA	TGGTTGTAGAGGGCAAGGAC
Lgals3	TGTGAAGGTGATGGGATGAG	AAGTGGTCGCTGAAGAGCAG
Gapdh	GACCCCTTCATTGACCTCAAC	GATGACCTTGCCCACAGCCTT
**Human**		
PPARA	TGGACACAGAGGAAAGAGCC	TGGCATCTTGTTGAAGGATGG
CPT1A	GCTGCTCCTGAGTCATGCTG	GGAGGACTTGGACACGAAGA
CPT2	GCAGATGATGGTTGAGTGCTCC	AGATGCCGCAGAGCAAACAAGTG
ACADC	GAGGAGGAGGAGAAGGAGGC	GTCATCCTCCAGCTCCTCCT
ACAD9	GGCTGTGAGGACGTTGACTT	TGGTCGATGTCAGGTTGTCT
ACAA2	AGGTGGTGGAAGAGGACTTT	CTTCTGCCTTCATCTGGTGA
SREBFC1	ACTTCTGGAGGCATCGCAAGCA	AGGTTCCAGAGGAGGCTACAAG
FASN	CCTGGAACCGTTATGAAGAT	ATGGCATGGTTGATGAGCTT
PPARG	CGAGAAGGAGAAGCTGTTGG	TCAGCGGGAAGGACTTTATG
DDIT3	AGCGACTTCAGGGTCGTTGCG	AGGCATGGAGCTGCAACGTCC
HSPA5	TAGCGTATGGTGCTGCTGTC	TGACACCTCCCACAGTTTCA
COL1A1	CCAAATCTGTCTCCCCAGAA	TCAAAAACGAAGGGGAGATG
ACTA2	GAGACCCTGTTCCAGCCATC	TACATAGTGGTGCCCCCTGA
PPARGC1A	CAGAGAGTATGAGAAGCGAGAG	AGCATCACAGGTATAACGGTAG
TFAM	ACCAAAAAGACCTCGTTCAGC	CGAGTTTCGTCCTCTTTAGCA
GAPDH	CCAAGGAGTAAGACCCCTGG	TGGTTGAGCACAGGGTACTT
